# Nanomaterials for Antiangiogenic Therapies for Cancer: A Promising Tool for Personalized Medicine

**DOI:** 10.3390/ijms22041631

**Published:** 2021-02-05

**Authors:** Hashem O. Alsaab, Alanoud S. Al-Hibs, Rami Alzhrani, Khawlah K. Alrabighi, Aljawharah Alqathama, Akram Alwithenani, Atiah H. Almalki, Yusuf S. Althobaiti

**Affiliations:** 1 Department of Pharmaceutics and Pharmaceutical Technology, Taif University, P.O. Box 11099, Taif 21944, Saudi Arabia; r.zhrani@tu.edu.sa; 2 Addiction and Neuroscience Research Unit, Taif University, P.O. Box 11099, Taif 21944, Saudi Arabia; ahalmalki@tu.edu.sa (A.H.A.); ys.althobaiti@tu.edu.sa (Y.S.A.); 3 Department of Pharmacy, King Fahad Medical City, Riyadh 11564, Saudi Arabia; aalhibs@kfmc.med.sa; 4 Batterjee Medical College for Sciences and Technology, Jeddah 21577, Saudi Arabia; 90102.Khwlah@bmc.edu.sa; 5 Department of Pharmacognosy, Pharmacy College, Umm Al-Qura University, Makkah 21955, Saudi Arabia; aaqathama@uqu.edu.sa; 6 Department of Laboratory Medicine, College of Applied Medical Sciences, Umm Al-Qura University, Makkah 21955, Saudi Arabia; aiwithenani@uqu.edu.sa; 7 Department of Pharmaceutical Chemistry, Taif University, P.O. Box 11099, Taif 21944, Saudi Arabia; 8 Department of Pharmacology and Toxicology, College of Pharmacy, Taif University, P.O. Box 11099, Taif 21944, Saudi Arabia

**Keywords:** angiogenesis, antiangiogenics, nanomedicine, theranostic, VEGF receptors, angiogenesis biomarkers

## Abstract

Angiogenesis is one of the hallmarks of cancer. Several studies have shown that vascular endothelium growth factor (VEGF) plays a leading role in angiogenesis progression. Antiangiogenic medication has gained substantial recognition and is commonly administered in many forms of human cancer, leading to a rising interest in cancer therapy. However, this treatment method can lead to a deteriorating outcome of resistance, invasion, distant metastasis, and overall survival relative to its cytotoxicity. Furthermore, there are significant obstacles in tracking the efficacy of antiangiogenic treatments by incorporating positive biomarkers into clinical settings. These shortcomings underline the essential need to identify additional angiogenic inhibitors that target numerous angiogenic factors or to develop a new method for drug delivery of current inhibitors. The great benefits of nanoparticles are their potential, based on their specific properties, to be effective mechanisms that concentrate on the biological system and control various important functions. Among various therapeutic approaches, nanotechnology has emerged as a new strategy for treating different cancer types. This article attempts to demonstrate the huge potential for targeted nanoparticles and their molecular imaging applications. Notably, several nanoparticles have been developed and engineered to demonstrate antiangiogenic features. This nanomedicine could effectively treat a number of cancers using antiangiogenic therapies as an alternative approach. We also discuss the latest antiangiogenic and nanotherapeutic strategies and highlight tumor vessels and their microenvironments.

## 1. Introduction

Cancer, which is characterized by irregular cell metabolism and metastasis risk development, remains a major and lethal risk to human life [1]. Although there are several unique benefits to cancer treatment, in recent years, problems such as poor targeting effectiveness, elevated tumor hypoxia, severe coronary syndromes, excessive ventricular conductivity, induced drug resistance, and increased risk of tumor metastases have limited their potential use in clinical settings [2,3,4,5].

**Figure 1:**
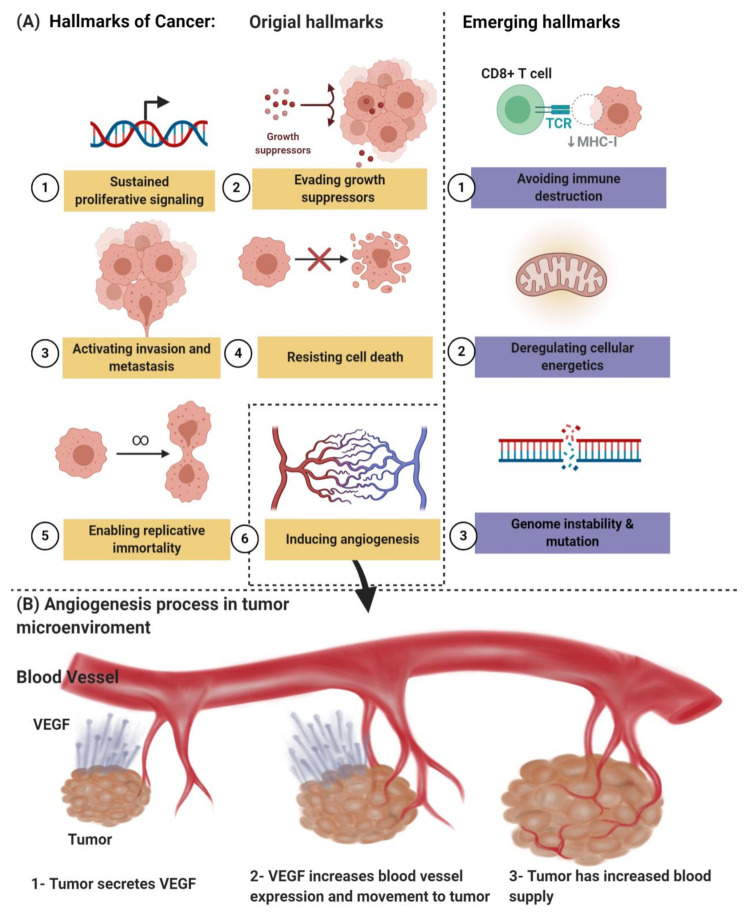
(**A**) The hallmarks of cancer include original hallmarks (sustained proliferative signaling, evading growth suppressors, activating invasion and metastasis, resisting cell death, enabling replicative immortality, and inducing angiogenesis) and emerging hallmarks (avoiding immune destruction and deregulating cellular energetics, genome instability, and mutation). (**B**) Angiogenesis process in tumor microenvironments. Created with BioRender.com.

Angiogenesis, which is one of the hallmarks of cancer, as shown in Figure 1A, is an early characteristic of tumor growth; therefore, an early diagnosis of the primary tumor and the emerging metastatic lesions is likely to be aided by molecular angiogenesis images. On the other hand, it has led to a considerable research and development effort by numerous academic and industrial groups, recognizing that inhibition of neovascularization can delay progression and perhaps even starve tumors to death. The “angiogenesis” term is usually used interchangeably with the word “neovascularization” [6,7]. Due to these efforts, a series of treatments, commonly called antiangiogenic medicine, was approved for clinical use. Several hundred late-stage clinical studies are underway for antiangiogenic medicinal products and combination regimens. Unfortunately, only relatively small, unexpected subsets of patients are affected by the anticancer drugs known as bevacizumab, sunitinib, sorafenib, and pazopanib, among others. This treatment may result in serious adverse events [2,6,7,8,9]. Together with the high cost of antiangiogenic medicine, these weaknesses have prevented their widespread acceptance by regulatory bodies and private and national insurance providers. Therefore, imaging-based methodologies are urgently required to identify new responders early and reliably in order to be used to refine and “personalize” antiangiogenic regimes that are image-guided.

Angiogenesis is an important condition for tumor growth, and it is considered a primary target for cancer treatment. Molecular angiogenetic imaging will effectively have the potential for diagnosing, improving, and controlling image therapy outcomes [10]. Innovations in micro-nanotechnology and cancer biology have facilitated the production of drug delivery systems with improved efficiency and reduced side effects for cancer treatment. The codelivery of antiangiogenic cancer therapeutics was made possible with a view to decrease drug side effects [11], increase target effectiveness [12,13], and enhance the stability and half-lives of nanomaterials based on natural/synthetic polymers [14,15], liposomes [16], metal–organic frameworks (MOFs) [17], or gold nanoparticles [18] and silica NPs [19,20]. This paper reviews the latest attempts to exploit drug delivery systems focused on nanomedicine for cancer angiogenesis biomarkers, focusing on the main multimodal imaging and antiangiogenic synergistic treatment strategies. These formulations illustrate both the design principles and their anticancer results. Finally, we discuss the challenges and development directions in this field.

## 2. Angiogenesis Pathways and Biomarkers

### 2.1. Angiogenesis Pathways in Cancer

Pathologic angiogenesis, which is defined by the creation of abnormal blood vascular networks within tumors due to an imbalance of pro- and antiangiogenic signaling, is considered one of the main hallmarks of cancer, as shown in Figure 1B. Elevated pressure of the interstitial fluid in the tumor and heterogeneity in tumor blood flow are the main physiological consequences of vascular abnormalities that fuel the tumor’s progression and contribute to therapeutic resistance to chemotherapy, radiotherapy, and immunotherapy. The discovery of the vascular endothelial growth factor (VEGF) as a significant driver of tumor angiogenesis has had its impact on united efforts to discover novel inhibitors against VEGF, with the hope of regressing tumors by starvation [21].

**Figure 2:**
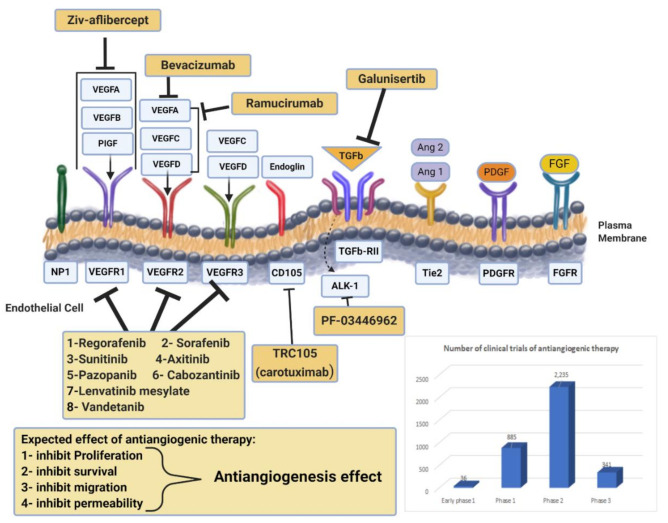
Drug targeting angiogenesis. VEGF-axis dependent and non-VEGF-mediated mechanisms of resistance to antiangiogenic therapies. Non-VEGF axis receptors: TGF-β receptor, Tie2, PDGFR, and FGFR. Antiangiogenic drugs mechanisms of action for anti-VEGF (monoclonal antibodies, RTK inhibitors) and novel targeted therapies are presented. Additionally, the chart represents the number of clinical trials of antiangiogenic therapy at all different phases. Data obtained from https://www.clinicaltrials.gov/ (accessed on 4 February 2021).

VEGF, which is overexpressed in many human cancers, is a predominant regulator of angiogenesis complex processes. As shown in Figure 2, the VEGF family has five members (VEGF-A, VEGF-B, VEGF-C, VEGF-D, and placental growth factor (PIGF)). These five ligands interact with three receptors (VEGFR-1, VEGFR-2, VEGFR-3). The interaction between VEGF-A and VEGFR-2 triggers endothelial cell migration and cell mitogenesis, leading to cancer development and metastasis. The interaction between VEGF-B and VEGFR-3 mainly maintains the newly formed blood vessels. However, VEGF-C and VEGF-D bind to VEGFR-3 and is primarily expressed in lymphatic vessels. Thus, VEGFR-3 and its ligands play a central role in lymph angiogenesis and the cancer cell spread to lymph nodes. PIGF is a cytokine that plays multiple roles in angiogenesis, including fueling tumor growth through the activation of stromal cells, myeloid cells, and bone marrow-derived endothelial progenitors. Neuropilin 1 (NRP1), neuropilin 2 (NRP-2), and heparan sulfate proteoglycans are identified as VEGF coreceptors. In addition, other cell surface receptors, like growth factor receptors and integrins, can crosstalk with VEGF. Moreover, the activity of VEGFR-2 can be induced by NRP-1 and NRP-2; however, these neuropilins can also signal independently.

The role of VEGF signaling in cancer has been documented by several studies [21,22,23,24]. Multiple reports have linked VEGFR-1 and VEGFR-2 to cancer progression events like cancer cell proliferation and metastasis. Indeed, human ovarian cancer has shown a functionally active VEGFR-2 and suggests that the efficacy of VEGF-targeted therapies may be mediated by antiangiogenic effects [25].

### 2.2. Antiangiogenic Biomarkers

**Figure 3:**
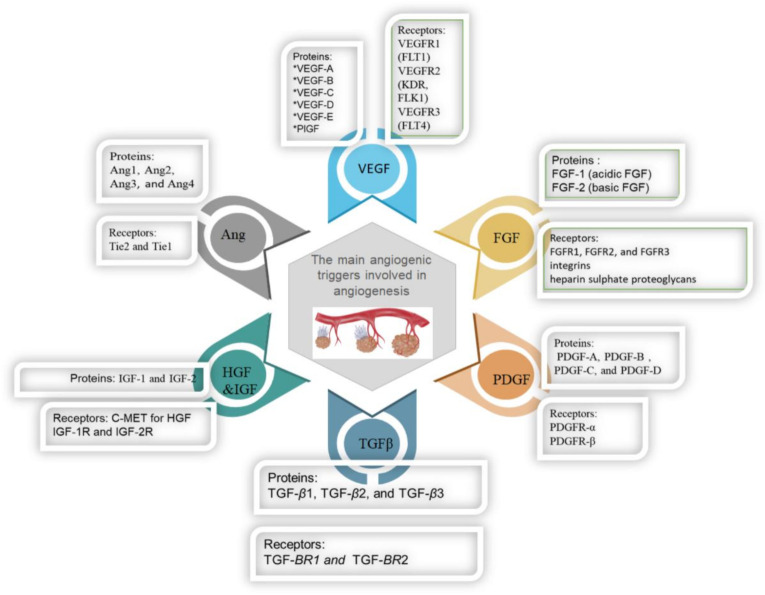
The main angiogenic triggers involved in angiogenesis.

In recent decades, studies have shown that the functional and molecular structures of angiogenic tumors versus normal vasculature have varied significantly. Several proteins are overexpressed in angiogenic vasculature at higher amounts on the surface of the cells and can serve as sufficient imaging targets, as presented in Figure 3 [26]. Many researchers have explored the targeted imaging of important biomarkers of tumor angiogenesis, integrins, and VEGF receptors. Molecular imaging of responses of model tumor systems to antiangiogenic therapy has shown an intricate pattern of targeted tracer build-up changes in tumors that represent drug-induced tumor rebounds after vascular recovery. Further studies also have evaluated the competitiveness of selective imaging of key markers for angiogenesis in early diagnosis and image-guided therapy [27,28]. Notably, these targets are accessible from the circulating blood, unlike biomarkers in tumor cells, so that tracer extravasation and tumor penetration can be easily imaged.

#### 2.2.1. Integrins

Integrins, particularly αvβ3 and αvβ5, are a group of angiogenic biomarkers. Integrins are transmembrane proteins known to be involved in growth, survival, adhesiveness, and motility, which are used as protein receptors in the extracellular matrix (ECM) and some superfamily immunoglobulins [29,30,31]. However, integrins are also expressed on several tumor cells in addition to the endothelial cells in angiogenic vasculature, and this should be taken into consideration in any experimental findings associated with integrin. One example of this is RGD (arginine-glycine-aspartic acid), which is found in several ECMs and some associated proteins, such as fibronectin, vitronectin, fibrinogen, laminin, collagen, von Willebrand factor, osteopontin, and thrombospondin.

#### 2.2.2. VEGF

The receptors of vascular endothelial growth factors (VEGFs) are another biomarker category overexpressed in the vasculature. VEGF is a crucial angiogenesis regulator, and its activity on endothelial cells is facilitated by two tyrosine kinase receptors, VEGFR-1 and VEGFR-2 (mainly VEGFR-2) [32,33]. VEGFR-2 is primarily expressed in endothelial cells, even though it can also be detected in other cells. In an immunohistochemical study, an endothelial group of cells is found to express significantly higher levels of VEGFR-2 than quiescent endothelial cells at angiogenesis sites, especially in tumor growth areas. Therefore, a VEGFR target for molecular diagnostic imaging is particularly advantageous.

The VEGF/VEGFR route is the primary target for antiangiogenic drugs because of its fundamental physiological relevance. The FDA has now approved the first blockbuster drug to treat multiple cancers for VEGFR; there are around 275,000 cases a year in the United States [34,35].

In tumor vasculature, many other receptors such as matrix metalloproteinases (MMPs), prostate-specific membrane antigen (PSMA), endogline (CD105), endosialin (CD248/TEM1), electric selectin, ECM components such as extra fibronectin domain B, and additional tenascin C domain are selectively overexpressed [36]. Besides integrins and VEGF receptors, many other proteins such as matrix-metalloproteinase (MMP), PSMA, endoglin (CD105), endosialin (CD248/TEM1), and ECM components are also selectively overexpressed in tumor vasculature.

### 2.3. Importance of Angiogenesis Biomarker Imaging

There is a massive opportunity to treat all major forms of cancer, with 12 million cases per year in the US, according to many hundreds of Phase III clinical trials (www.clinicaltrials.gov (accessed on 4 February 2021)). However, as described above, individual patients have a complicated and unreliable response to antiangiogenic drugs and combination treatments. In this regard, it could be useful for therapy optimization to track VEGF receptor prevalence in the initial response to VEGF/VEGFR-targeted medications.

Currently, selective tracers are fairly well-developed for molecular visualization of integrins and VEGF receptors; some RGD-based tracers are also in clinical trials. Only systematic clinical research can assess whether imaging using such molecular tracers will be able to diagnose the stage of primary tumors and metastatic lesions with MRI, CT, or metabolic PET imaging. Molecular imaging for image-driven therapy may be more beneficial, with results tested in real-time in actual patients by medicines directly targeted at integrins, VEGF receptors, or other targets in the angiogenic vasculature. This strategy might entail thorough clinical trials and multidisciplinary cooperation as this strategy could lead to resolving unaddressed medical needs, given the increasing demand for personalized but also not-too-expensive medicines.

## 3. Clinical Trials in Antiangiogenic Therapeutics

Antiangiogenetic therapy has become of great interest in cancer treatment in addition to the conventional therapies of chemotherapy and radiation. Targeting this hallmark of cancer progression leads to the prevention of new blood vessel development and the eradication of existing tumor blood vessels. Inhibiting angiogenesis in metastasis compromises the blood supply to tumor cells, depriving them of nutrients and preventing further growth [37,38]. Antiangiogenetic therapy may also reduce the degree of tumor malignancy by alleviating hypoxia levels within the tumor microenvironments and improving the efficacy of conventional approaches [38]. The development of blood vessels within tumors occurs when the proangiogenic factors (e.g., VEGF, basic fibroblast growth factor (bFGF), hepatocyte growth factor (HGF), insulin-like growth factor 1 (IGF-1), and angiopoietin-2), and the antiangiogenic factors (e.g., thrombospondin-1 and angiopoietin-1) are out of balance. Different approaches to targeting angiogenesis have been tested in clinical practice, including monoclonal antibodies binding VEGFs (such as bevacizumab), RTK inhibitors (like sunitinib and sorafenib), and mTOR protein inhibitors that mediate VEGF signaling (such as everolimus) [39]. Antiangiogenic therapies that have been approved by the FDA are summarized in Table 1, with examples discussed below, while active clinical trials for those drugs are counted in Figure 2.

The US FDA has approved several antiangiogenic agents for cancer treatment; these include monoclonal antibodies [40] that target specific proangiogenic growth factors and their receptors (ramucirumab and bevacizumab), tyrosine kinases inhibitors (TKIs; axitinib, sunitinib, sorafenib, regorafenib, cabozantinib, pazopanib, and vandetanib) and inhibitors of mammalian target of rapamycin (mTOR; everolimus and temsirolimus). Despite their availability, many of these agents have limited clinical uses [41].

### 3.1. Selected Examples of FDA Approved Antiangiogenic Agents

#### 3.1.1. Bevacizumab (Avastin^®^)

The first inhibitor of angiogenesis to be approved by the FDA was bevacizumab (Avastin), a monoclonal antibody used to treat colorectal cancer that binds to VEGF-A and inhibits interaction with their receptors. This suppresses the VEGF signaling pathways and blocks angiogenesis. Initial approval was given in the USA (2004) and the European Union (2005) to treat different solid tumors. Clinical efficacy has been proven in metastatic colorectal cancer (mCRC), nonsmall-cell lung cancer (NSCLC), glioblastoma multiforme (GBM), renal cell carcinoma (RCC), metastatic breast cancer, and ovarian cancer [42].

In a phase III clinical trial involving 813 cases of previously untreated mCRC, 402 patients were given irinotecan, bolus fluorouracil, and leucovorin (IFL) plus bevacizumab, and 411 were given IFL plus a placebo. Findings showed improved survival duration in the bevacizumab group (20.3 months vs. 15.6 months) and a corresponding low hazard ratio for death (0.66). In addition, both progression-free survival (10.6 months vs. 6.2 months) and response duration (10.4 months vs. 7.1 months) increased [43]. In another phase III trial (NCT00021060) of 878 patients with recurrent or advanced NSCLC, 434 were given paclitaxel and carboplatin plus bevacizumab, and 444 were given paclitaxel and carboplatin alone. The progression-free survival rate was higher in the group treated with bevacizumab (6.2 vs. 4.5 months), with a rise in median survival (12.3 months vs. 10.3 months) and a 0.79 death hazard ratio. Clinically significant bleeding rates in the two groups, one group treated with chemotherapy plus bevacizumab, and the other with chemotherapy alone, was 4.4% and 0.7%, respectively [44].

Bevacizumab, in combination with paclitaxel, was approved by the FDA in 2008 for metastatic breast cancer. However, further trials showed no significant improvement in overall survival, and FDA approval for metastatic breast cancer was withdrawn in 2011 [45]. Bevacizumab combined with interferon-alpha 2A and interferon-alpha 2B for metastatic melanoma has been investigated and showed clinical response in 24% of patients with stage IV melanoma [46]. Later, the FDA approved it in 2014 and 2018 as part of combination therapies using bevacizumab with paclitaxel and cisplatin or with paclitaxel and topotecan for persistent, recurrent, or metastatic cervical cancer; bevacizumab in combination with carboplatin and paclitaxel was also approved for ovarian cancer [45,47].

Furthermore, using bevacizumab in the modulation of tumor-induced immunosuppression expands the possibilities of its role in immunotherapy combinations, which have been investigated in clinical trials. Different combinations of bevacizumab and immunotherapy have been approved for solid tumors. In 2020, the FDA approved atezolizumab (PDL-1 inhibitors) in combination with bevacizumab for unresectable or metastatic hepatocellular carcinomas [48]. A phase III trial (NCT03434379), in which 336 patients were given atezolizumab plus bevacizumab and 165 patients given sorafenib, resulted in better overall and progression-free survival outcomes for the atezolizumab/bevacizumab group than the sorafenib group (6.8 months vs. 4.3 months) [49].

#### 3.1.2. Sunitinib (Sutent^®^)

Sunitinib is an RTK inhibitor that was approved by the FDA in 2006 for gastrointestinal stromal tumor (GIST) and in 2007 for advanced RCC, based on clinical investigations showing remarkable objective response rates and clinical benefits [50,51]. It selectively inhibits four RTKs that play major roles in angiogenesis—VEGFR-2, platelet-derived growth factor receptor-β, fibroblast growth factor receptor 1, and epidermal growth factor receptor—thus targeting the VEGF-signaling pathway [50].

After sunitinib demonstrated its efficacy in GIST in phase I/II trials, a phase III trial was conducted with a total of 302 patients receiving either sunitinib (n: 207) or a placebo (n: 105). The median time to progression for the sunitinib group was 27.3 weeks compared to 6.4 weeks for the placebo group. However, overall survival did not change significantly in either the sunitinib or the placebo group (73.9 weeks vs. 64.9 weeks). Sunitinib, in combination as an adjunct to second- and third-line FOLFIRI in chemotherapy-resistant gastric cancer, was evaluated in a phase II trial (NCT01020630). The progression-free survival and response rates did not improve, although a trend towards better overall survival was observed in the FOLFIRI + sodium folinate + sunitinib group [52].

Sunitinib is also the first-line treatment for mRCC. A phase III trial (NCT00098657 and NCT00083889) involving 750 patients with untreated mRCC showed a higher response rate in those treated with sunitinib compared to the interferon alfa group. Progression-free survival was longer in the sunitinib group (11 months vs. 5 months), along with a remarkably higher objective response (31% vs. 6%). In addition, grade 3 or 4 treatment-related fatigue was reported to be greater in the interferon alfa group compared to the level of diarrhea side-effects in the sunitinib group [53,54]. Sunitinib was also approved for pancreatic neuroendocrine tumors (NETs) by the FDA in 2011 after a phase III trial (NCT00428597), in which 171 patients with advanced, well-differentiated NETs that were given sunitinib alone showed longer progression-free survival (11.4 months vs. 5.5 months) than a placebo group. Overall survival and objective response rate survival (9.3% vs. 0%) were also improved in the treated group, with 10% deaths compared to 25% in the placebo group [55].

#### 3.1.3. Everolimus (Afinitor^®^)

Everolimus inhibits mTOR proteins, which are multifunctional signal-transducing proteins that work downstream of different signaling pathways and affect protein translation in cancers. Everolimus inhibits tumor growth via its influence on VEGF levels; it was approved for solid tumor types in May 2009 [56]. Phase I, II, and III trials have shown clinical efficacy, and the drug has been used as a second-line treatment in RCC, BC, and NET [57].

A placebo-controlled phase III trial was conducted in a group of 410 patients with mRCC. One group of 272 patients was given 10 mg everolimus daily, compared with a placebo group of 138 patients. The primary endpoint was median progression-free survival, which was higher in treated patients (4.0 vs. 1.9 months; hazard ratio 0.30, 95% confidence interval 0.22–0.40). No significant difference in overall survival was recorded, but this was mainly the result of 80% of the placebo group switching over to everolimus treatment [58]. In a phase III trial (NCT00510068) of 410 patients with low or intermediate grade NETs that had progressed in the last year, 207 received 10 mg everolimus daily and 203 were given a placebo. The total progression-free survival rate doubled in the treated group (11 months vs. 4.6 months), and, at 18 months, higher rates were recorded of around 34% (95% CI: 26 to 43) in the everolimus group compared to 9% (95% CI: 4 to 16) in the placebo group [59].

The FDA also approved everolimus in 2012 to treat postmenopausal women with hormone-receptor-positive advanced BC and human epidermal growth factor receptor 2 (HER2)-negative type BC. In a BOLERO-2 phase III clinical trial (NCT00863655) involving 724 patients with progressed or recurred hormone-receptor-positive advanced breast cancer, one group was given everolimus combined with an aromatase inhibitor, and the other group was given placebo plus an aromatase inhibitor. Longer median progression-free survival was detected in the group treated with everolimus plus exemestane compared to the placebo plus exemestane group (6.9 months vs. 2.8 months; hazard ratio (HR): 0.36; 95% CI: 0.27 to 0.47) [60].

A TAMRAD phase II trial treated a group of 111 metastatic BC patients who were HR-positive/HER2-negative and had had prior exposure to aromatase inhibitors. Everolimus plus tamoxifen was given to 54 of the women; the remaining 57 received tamoxifen alone. Average times to progression increased to 8.6 months with tamoxifen plus everolimus compared to 0.5 months with tamoxifen alone, indicating a 46% reduction in risk of progression with tamoxifen plus everolimus (hazard ratio: 0.54; 95% CI: 0.36 to 0.81). In addition, a reduction of death risk to 55% was shown for the combination treatment (HR: 0.45; 95% CI: 0.24 to 0.81) [60].

### 3.2. Angiogenesis Inhibitor Challenges

Although antiangiogenic drugs have shown undeniably positive activity in clinical practice, they have also revealed detrimental challenges in some cases. Adverse complications associated with angiogenesis inhibitors have been reported, such as hemorrhage, endocrine dysfunction, thrombosis, hypertension, cardiac toxicity, proteinuria, and reversible posterior leukoencephalopathy. Furthermore, some patients on VEGF inhibitors have had to receive anticoagulant treatments due to higher thromboembolism risks of up to 5%. Increased risk of hypertension has been seen in 25% of patients on this regimen, as well as uncontrolled hypertension linked to further adverse effects such as reversible posterior leukoencephalopathy and proteinuria, which occasionally leads to a permanent cessation of VEGF inhibitor therapy and, consequently, causes protein reduction (up to 3 g protein loss in 24 h) [61]. Although bevacizumab treatment with paclitaxel plus carboplatin in NSCLC has shown significant positive clinical outcomes, febrile neutropenia and pulmonary hemorrhage have sometimes been reported as a result of anti-VEGF treatment [44].

In some cases, tumors treated with antiangiogenic agents have demonstrated several different forms of innate and acquired resistance mechanisms, pointing to this therapy’s possibly limited clinical significance. Resistance is an area that would benefit from further research since it occurs via a range of mechanisms, including VEGF-dependent alterations, alternative growth-factor signaling pathways, and stromal cell interactions [62]. One developed novel mechanism of resistance towards sunitinib is lysosomal sequestration, which prevents drug penetration to the kinase domain of RTK present in the cytoplasm and diminishes drug potency [63]. Another means of tumor cell escape from antiangiogenic drugs lies in revascularization in a hypoxic microenvironment, which works via upregulation of proangiogenic signals or vasculogenic mimicry, leading to the protection of vasculature in the tumor; this latter effect has been reported in bevacizumab treatment [64,65].

A biomarker-dependent way of selecting cancer patients for antiangiogenic therapy needs to be approved, with a considerable number of approved angiogenesis inhibitors available. Well-established biomarkers are lacking in the areas of efficacy monitoring, safety, resistance to VEGF-targeted therapy prediction, and cost considerations. Developing an effective biomarker system would help to personalize antiangiogenic therapy for each patient and, thus, increase its chances of success [66]. The current challenges associated with antiangiogenic drugs, such as increasing bioavailability, minimizing toxicity, and overcoming resistance, need to be rapidly addressed to facilitate treatment decisions.

## 4. Utilizing Nanomedicine for Antiangiogenic Medication

A new form of treatment could open a new frontier for cancer therapy by targeting angiogenesis. Among various therapeutic approaches, nanotechnology has emerged as a new strategy for the treatment of different cancer types [67]. Nanoparticles (NPs) are nanosized materials that can deliver high therapeutic doses into tumor cells without affecting healthy cells. Thus, NPs can solve the limitations of traditional strategies such as the impact on normal cell replication and deviation, unwanted side effects, and drug resistance [68]. In the next sections, we focus on recent efforts to utilize nanomedicine-based drug delivery systems for cancer angiogenesis biomarkers and focus on the new ways of multimodal imaging and synergistic antiangiogenic treatments.

### 4.1. Nano-Antiangiogenic-Based Cancer Monotherapy

Excessive production of angiogenic stimulators such as VEGF may trigger different types of malignancies [38]. Therefore, VEGF and EGFR inhibitors have a significant role in various tumor types. Combining different therapeutic inhibitors to target different signaling pathways can be more effective than single pathway inhibitors [69]. The antiangiogenic agents are currently manufactured to prohibit tumor cells from receiving nutrients by hindering new vessel formation and extirpating the available ones. Inhibition of PDGF and VEGF-receptor (VEGFR) can block VEGF signals by using small molecules such as tyrosine kinase inhibitors (TKIs). Antiangiogenic agents such as aflibercept and bevacizumab showed significant activity when combined with cytotoxic agents [70]. However, vasculature can be targeted by therapeutic NPs, which can be optimized by conjugating VEGF-2 targeted ligands such as an antibody. Thus, different NP-based antiangiogenic drug delivery systems have been well established by researchers, including lipid nanoparticles [71], gold [72,73], silver nanoparticles [74], and silica- and silicate-based nanoparticles [75].

Silica- and silicate-based nanoparticles have been utilized for antiangiogenic cancer therapy. Setyawati et al. recently indicated that restrictions in endothelial cell proliferation, invasion, and migration eventually impede a signaling cascade that causes tumor growth. This cascade results from intracellular reactive oxygen species production, which activates the p53 tumor suppressor pathway, caused by size-dependent antiangiogenic therapy of mesoporous silica nanoparticles. Moreover, Mukherjee et al. showed the antiangiogenic property of 5 nm of spherical bare gold nanoparticles (AuNPs) for the first time. Thus, gold NPs have been used in nanomedicine due to their biocompatibility and high drug loading. Recently Pan et al. observed that AuNPs could inhibit tumor angiogenesis by inhibiting AKT and VEGF165, which induce VEGFR2 phosphorylation [76].

### 4.2. Synergistic Antiangiogenetic Activity with Chemotherapy

The search for effective cancer treatment continues [11]. Using combination therapies to inhibit signaling pathways can achieve greater efficiency than targeting a single pathway. EGFR and VEGF inhibitors are the key therapy in many tumor types [69]. Thus, using nanocarriers is one of the viable methods to deliver chemotherapeutic agents and small molecules [77]. Angiogenic inhibitors can be loaded on/within nanocarriers by encapsulation or chemical conjugation, to be delivered passively or actively to the tumor cells. A study showed that nanoparticle-conjugated chemotherapeutic agents such as doxorubicin (DOX) and small antiangiogenic molecules could preferentially home in on tumors using an enhanced permeability and retention effect (EPR) that results in tumor growth inhibition and selective vascular shutdown [78,79].

Furthermore, bevacizumab (an FDA-approved anti-VEGF recombinant humanized immunoglobulin G1 monoclonal antibody) is widely used in combination with 5-fluorouracil (5-FU) and irinotecan-based chemotherapy regimens for colorectal cancer as a first-line treatment based on randomized, controlled clinical trials (RCTs) that have shown survival benefits over chemotherapy alone. Moreover, bevacizumab also showed survival benefits as a second-line treatment for advanced colorectal cancer when added to oxaliplatin-containing chemotherapy [69]. It has been found that mitomycin C (MMC) and DOX coloaded, polymer–lipid hybrid nanoparticles can significantly improve the tumor cure rate and animal survival in comparison with liposomal DOX for multidrug-resistant human mammary tumor xenograft treatment. Another potent antiangiogenic agent is curcumin (Cur) that was coloaded with DOX into pH-responsive poly-(beta-amino ester) copolymer NPs for tumor 4T1 treatment, which has intensive proapoptotic and antiangiogenic activities [80].

### 4.3. Synergistic Antiangiogenic Activity with Gene Therapy

As new therapeutic gene suppression technology, small interfering RNAs (siRNAs) have attracted much attention compared to other therapies [81]. Gene therapy introduces nucleic acids such as DNA, miRNA, or siRNA as the drug, and it has certain advantages. It is another potential therapeutic strategy to inhibit tumor growth [82]. This approach allows siRNAs to specifically bind to their target mRNAs, which leads to cleavage and gene suppression [81]. The therapeutic gene has seen much evolution in two parallel paths: viral and nonviral. The viral gene delivery method has a significant obstacle due to the potential toxicity of certain viruses and immunogenicity.

On the other hand, nonviral gene delivery nanoparticles are utilized by both natural and synthetic lipids and polymers [82]. It is well established that inhibiting angiogenesis via nanoparticle-delivered genes are much safer to synthesize, less toxic, and easier than their “viral vector” counterparts. Hood and coworkers’ study combined lipid nanoparticles and αvβ3-targeting moiety (the LM609 antibody); these NPs carry a mutant Rag gene, ATPμ-Raf-1. NPs deliver the gene to tumor vasculature and interfere with the signaling cascades of two important key roles of angiogenic growth factors—basic fibroblast growth factor (bFGF) and VEGF [83]. Another study demonstrated a synergistic effect when combining VEGF-targeted RNAi and suicide gene therapies. This combination could significantly suppress the tumor growth of SGC7901 xenografts in mice and effectively kill SGC7901 cells in vitro. The finding showed that tumor cell apoptosis could be induced more effectively by codelivering VEGF siRNA using calcium phosphate nanoparticles (CPNPs) with a suicide gene (yCDglyTK) [84]. In 2017, Kim and coworkers demonstrated that poly-VEGF siRNA could form stable nanoparticles with thiolated-glycol chitosan via chemical bond formation and charge interaction. Therefore, tumor growth and VEGF gene expression suppression are obtained due to psi (VEGF)/tGC NP accumulation in the tumor. However, psi (VEGF)/tGC NPs and bevacizumab have a synergistic effect when combined. They can improve therapeutic outcomes and overcome bevacizumab resistance in cancer therapy [81].

### 4.4. Synergistic Antiangiogenetic Activity with Immunotherapy

Nanomedicine can be utilized to promote the induction of immunogenic cell death (ICD) by using doxorubicin-loaded liposomes (Caelyx/Doxil) combined with immunotherapy. Rios-Doria and colleagues published a study that combined DOX with different clinically relevant immunotherapeutic anti-PD-1, PD-L1, and -CTLA4 antibodies and tumor necrosis factor receptor alpha agonists. DOX improved immunotherapy efficacy by promoting DCs and CD8+ T-cell proliferation via ICD [85]. Thus, immune-checkpoint inhibitors are gaining a lot of attention in oncology [86]; their combination with antiangiogenic agents may improve cancer patients’ outcomes [87].

### 4.5. Synergistic Antitumor Microenvironment Agents/Photodynamic Therapy

PDT is a promising strategy currently used to tackle many malignancies with minimum invasiveness, fewer side effects, and a shorter treatment period than the conventional chemotherapeutic agents [88]. The tumor microenvironment (TME) is one of the main challenges in the cancer environment; it is where cancer cells interact with different cellular elements such as the extracellular membrane (ECM), endothelial cells, mesenchymal stromal cells, and cancer-associated fibroblasts (CAFs). These interactions play a significant role in complicating tumor therapy and enhance its progression and metastasis [89,90]. It is well known that ECM is one of the TME obstacles that builds up a dense barrier that attenuates drug diffusion into the tumor site. Liu et al. developed a TME nano-responsive nanoparticles encapsulated with collagenase composed of both Mn+^2^ and benzoic-imine linker. The nanostructures were further modified by PEG to enhance biological biocompatibility. The current nanostructure is a pH-sensitive nanostructure that decomposes and releases collagenase within the TME and breaks the TME–collagen linkage. Liu et al. found that breaking the collagen linkage improved the efficacy of PDT therapy [91]. Another challenge of the cancer TME is hypoxia, which plays a significant role in oxygen-dependent cancer therapies [92]. PDT functions by converting tumor oxygen to reactive singlet oxygen (ROS), which is able to activate the photosensitizer [93]. However, inadequate oxygen within the tumor site attenuates PDT efficiency. Thus, perfluorocarbon (PFC) nanoparticles is an interesting oxygen nanocarrier that is characterized by their biocompatibility and their ability to dissolve gas (O_2_, NO, and CO_2_) in a predictable manner [94,95]. Cheng et al. were able to develop lipid nanoparticles encapsulated with oxygen-enriched PFCs (LNO-PFCs). LNO-PFCs were fabricated to provide sufficient intratumor oxygen to the photosensitizer (IR780). Cheng et al. found that LNO-PFCs were able to provide oxygen for a long time and enhanced the overall PDT effect [96].

## 5. Significance of Antiangiogenics-Based Theranostic Agents and Possible Mechanisms

**Figure 4:**
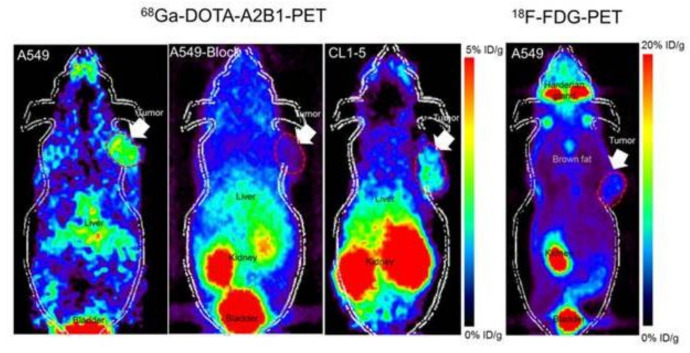
Noninvasive PET imaging of 68Ga-DOTA-A2B1, with/without a blocking dose of c(DGEAyK) peptide and 18F-FDG, in integrin α2β1-positive A549 and CL1-5 xenograft mouse models. The quantified PET imaging data indicates the binding specificity and favorable biodistribution pattern of integrin tracers. The tumors are indicated with arrows. Reproduced from [103].

Angiogenesis is an essential condition for the growth of tumors; therefore, it is a primary goal in the treatment of cancer. Molecular angiogenesis imaging offers new potential to initially diagnose and to optimize and manage therapeutic outcomes with images [97,98,99,100]. Many studies have focused on the developments of targeted imaging of essential tumor angiogenesis biomarkers, integrins, and VEGF receptors. Tracers for targeted imaging of these biological markers are now relatively well-developed in various imaging modalities, and PET tracers for integrin imaging are currently under investigation, as presented in Figure 4 [101,102,103,104]. A complex pattern of targeted tracer accumulation changes in tumors, reflecting drug-induced tumor regression following a vascular rebound, has been demonstrated through molecular imagery of longitudinal responses of model tumor systems to antiangiogenic therapy [105].

**Figure 5:**
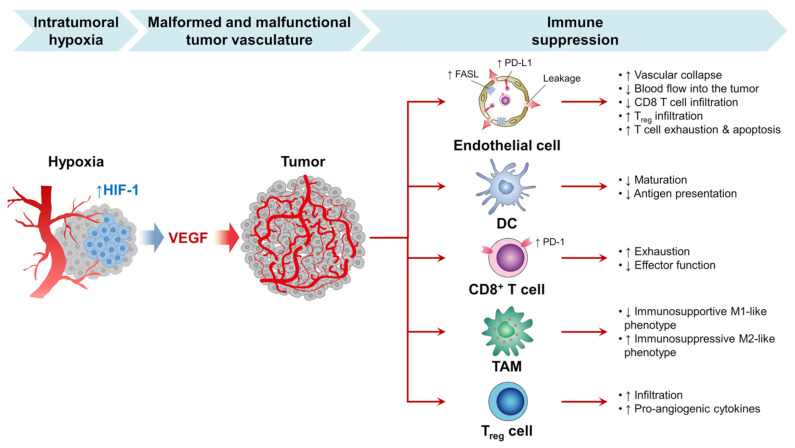
The growth of the tumor is dependent on sufficient blood vessel oxygen and nutrients. However, tumor development also exceeds current vascular supplies in rapidly progressing tumors and contributes to intratumor hypoxia. Hypoxia activates the angiogenic master switch, called the hypoxia-inducible factor-1 (HIF-1), and upregulates vascular endothelial growth factor (VEGF) in tumors. In turn, VEGF promotes tumor angiogenesis by inducing the proliferation and survival of endothelial cells (ECs), forming a myriad of malformed and malfunctional neovessels within the tumor. These tumor vessels interact with the selection of successful anticancer immunity in many phases and prohibit immune checkpoint inhibitors (ICI) therapy from being successful against the tumor [112]. Note: up and down arrows mean increase and decrease, respectively.

Within TME, one reason for the overstimulating release of VEGF is a hypoxic condition, as shown in Figure 5 [106,107]. Typically, angiogenesis is a biological balance between proangiogenic and antiangiogenic factors. However, in cancer cells, the angiogenetic factors are overstimulated to nourish cancer and maintain its growth and proliferation. Thus, using angiogenetic inhibitors is one of the strategies currently used to inhibit angiogenesis or disrupt the pre-existing tumor blood vessels [37]. Studies have shown that treating cancer cells with PDT therapy leads to VEGF upregulation [108]. Thus, combining both antiangiogenic inhibitors and PDT is a feasible way to enhance cancer therapy [109,110,111]. In another study, Min et al. successfully developed a porphyrinic nanostructure loaded with VEGF-R2 inhibitor (apatinib) and coated with MnO_2_. The surface of the nanoparticles was further decorated with the 4T1 cell membrane. MnO_2_ was utilized to deplete the cancer cell’s excessive production of GSH; thus, apatinib will be released from nanostructure to block PDT, inducing the angiogenic process [109].

**Figure 6:**
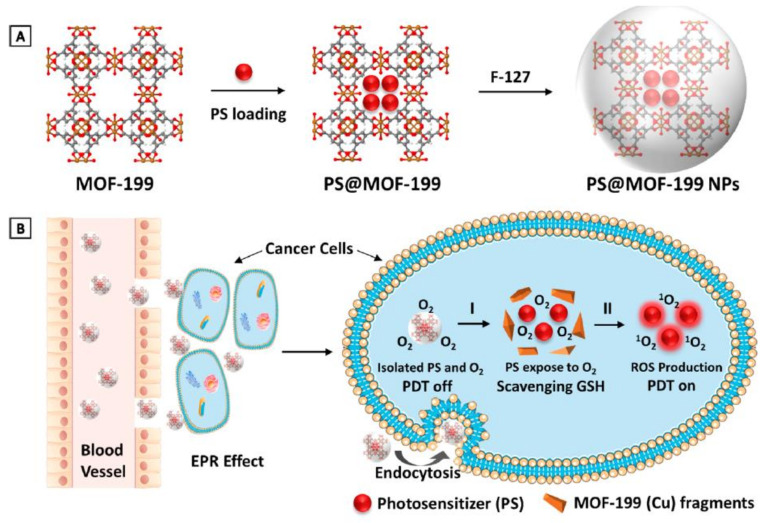
MOF-199, a Cu(II) carboxylate-based metal–organic framework (MOF), as an inert carrier to load PSs with prohibited photosensitization during delivery. (**A**) Synthetic scheme to PS@MOF-199 and F127-coated PS@MOF-199 (PS@MOF-199 NPs). (**B**) Quench and trigger of photosensitization originated from PS@MOF-199 NPs in the tumor microenvironment. Adapted with permission from [113].

Another mechanism that cancer cells use to overcome PDT therapy is the antioxidant defense mechanism to counteract cytotoxic ROS. Therefore, incorporating antioxidant depletion ligands or agents into a nanocarrier can overcome the cancer cells’ endogenous antioxidant defense mechanism; for instance, using nanoparticles containing high valent metal ions that can function as an antioxidant-depleting agent and a carrier for photosensitizers. Thus, Wang et al. used MOF-199, a Cu II carboxylate-based metal–organic framework (CuII-MOF), as an inert carrier for a photosensitizer agent (PS), as presented in Figure 6. Once the nanoparticle is endocytosed, Cu II reacts with endogenous cancer glutathione, leading to the suppression of the cancer antioxidant defense mechanism, releasing the PS within the TME, and consequently enhances PDT therapy [113].

In addition to the abovementioned PDT to overcome the cancer defense mechanism, PDT has been found to stimulate the suppressed immune system, as shown in Figure 5. Several nanoparticles strategies have been developed to enhance PDT’s TME immunosuppressive nature [93]. Chen et al. used a hemoglobin and HSA proteinaceous oxygen carrier loaded with a photosensitizer (Ce6). Hemoglobin plays a significant role in carrying the oxygen into the TME; upon laser activation, the cytotoxic oxygen species will be released and triggered an antitumor immune response. The dying cancer cells will enhance dendritic cell maturation and further activate natural killers and T-lymphocytes. Chen et al. found this nanocarrier was able to augment the immunogenic effect of PDT to eradicate primary tumor cells and inhibit its metastasis [114]. A wide variety of antiangiogenic–theranostic agents has been developed for the treatment of neoplasms [41,115,116]. Imaging studies play an important role in assessing these treatments’ effects [117].

## 6. Imaging Modalities Utilized for the Theranostic Purpose of Better Nanomaterials 

**Figure 7:**
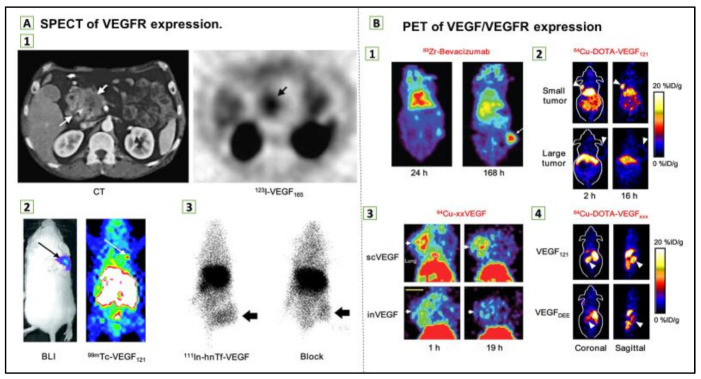
(**A**) Single-photon emission computed tomography (SPECT) of vascular endothelial growth factor receptor (VEGFR) expression. (1) Transverse CT image of pancreatic adenocarcinoma patient (**left**) and transverse SPECT image of the same patient at 1.5 h after injection of 123I-VEGF165 (**right**). (2) Bioluminescence imaging (BLI; after injection of d-luciferin) and SPECT images (after injection of 99mTc-VEGF121) of a tumor-bearing mouse. Tumor cells were transfected with firefly luciferase. (3) Posterior whole-body images of tumor-bearing mouse at 48 h after injection of 111In-hnTf-VEGF and after coinjection of 100-fold excess of unlabeled apotransferrin (block). Arrows in all images indicate tumors. (**B**) Positron emission tomography (PET) of VEGF/VEGFR expression. (1) Coronal small-animal PET images of a tumor-bearing mouse at 24 and 168 h after injection of 89Zr-bevacizumab. (2) Coronal small-animal PET images of U87MG tumor-bearing mice at 2 and 16 h after injection of 64Cu-DOTA-VEGF121. The small tumor expressed a high level of VEGFR-2, and the large tumor expressed a low level of VEGFR-2. (3) Coronal small-animal PET images of 4T1 tumor–bearing mice at 1 and 19 h after injection of either 64Cu-scVEGF (single-chain VEGF that binds to VEGFR) or the equivalent amount of 64Cu-inVEGF (inactive VEGF that does not bind to VEGFR). (4) Coronal and sagittal slices containing kidneys (arrowheads) at 4 h after injection of 64Cu-DOTA-VEGF121 (binds to both VEGFR-1 and VEGFR-2) or 64Cu-DOTA-VEGFDEE (VEGFR-2-specific). Arrows in 1–3 indicate tumors. Reproduced from [97].

Biomedical nanoparticles are being tirelessly developed and used due to their unique properties, confer by their modular structure, size, and functionalization abilities as shown in Table 2 [118]. Thus, molecular imaging permits cancer-related biomarker detection and visualization in tumors [119]. Superparamagnetic iron oxide nanoparticles (SPIONs), used for contrast generation with magnetic resonance imaging (MRI), were among the first nanoparticle structures to allow molecular imaging; however, MRI techniques have an advantage as a noninvasive method of functional, structural, and metabolic phenotype assessment of cancer on a variety of scales [120]. On the other hand, positron emission tomography (PET) and single-photon emission computed tomography (SPECT) are considerably used as noninvasive imaging modalities in clinical settings for oncology, as shown in Figure 7 [119]. PET detects gamma-ray pairs indirectly released by specifically labeled radionuclide tracers to provide metabolic or functional information in various disease scenarios. However, the most widely used imaging technique to study glucose uptake in tumors in-vivo is 8F-fluorodeoxyglucose (FDG) PET [120]. Numerous gold nanoparticle (AuNP) formulations have been sophisticated contrast agents for computed tomography (CT), which is considered as one of the most extensively used medical imaging methods. CT has the capability of producing highly temporal and spatial images at a relatively low cost [121].

## 7. Conclusions and Future Prospects

Angiogenesis is a targeted mechanism mediated by the VEGF and its receptors in a group of angiogenic factors. Given the wide-range use of antiangiogenic agents, significant interest has been shown in developing methods to detect new markers that can predict the effects of angiogenesis inhibitors for treating cancer conditions at different stages of development; these biomarkers include biomarkers of tissue, serum, and imaging. A major obstacle for biomarker exploration is the angiogenesis process’s sophistication and the overlap between the different angiogenic factors. Imaging biomarkers are quantitative imaging indicators capable of systematically explaining biological pathways, pathological changes, and therapeutic reactions in various contexts. The development and use of these image markers will overcome problems but can also facilitate the continued advancement of antiangiogenic therapy. In terms of substantial evidence, prospective studies remain necessary if the existing data is to be consolidated and novel biomarkers established. While many experiments have yielded promising findings, there is a lack of conclusive evidence. Long-term trials are needed to ensure that encouraging biomarkers in cancer patients are accurately predictive rather than qualitative. Without biomarkers, the decision to treat patients with an inhibitor of angiogenesis remains a therapeutic option on the basis of the balance between the benefit and the toxicity of antiangiogenic agents.

Together, attempts are continuing, notably in terms of cancer treatment, to effectively target pathologic angiogenesis while addressing existing limitations of angiogenesis inhibitors. Angiogenesis has certainly been influenced by the advancement of nanotechnology. Recently, several nanoparticles that demonstrate antiangiogenic properties have been developed and engineered. These nanomedicines may be effective for treating different cancers using antiangiogenic therapy. Theranostic nanoparticles allow the preselection of patients for optimum (nano-) chemotherapeutic formulation and, thus, tend to encourage the theory of personalized treatment. Finally, in order to continue promoting clinical translation for the diagnostics and therapy of the nanoparticles, particularly in the field of oncology, it is important to address the main regulatory challenges of nanoparticle synthesis regulation, uniformity, reproduction batch-to-batch, and upscaling nanoparticles. This is of extreme significance since batch-dependent variations in the size and form of nanoparticles have a significant effect on blood circulation, biodistribution, and nanoparticle removal. Finally, the latest attempts to exploit drug delivery systems have focused on nanomedicine for cancer angiogenesis biomarkers; the main multimodal imaging and antiangiogenic synergistic treatment strategies might be promising tools to personalize medicine for cancer patients.

## Figures and Tables

**Table 1:** Drug targeting angiogenesis pathways in phase IV clinical trials.

Clinical Trials Number	Intervention (Drug)	Cancer Type	Title of Study
NCT01525550	Sunitinib	Well-differentiated Pancreatic Neuroendocrine Tumor	A Study of The Efficacy and Safety of Sunitinib In Patients with Advanced Well-Differentiated Pancreatic Neuroendocrine Tumors
NCT02582970	5-Fluorouracil Bevacizumab Irinotecan Oxaliplatin	Colorectal Cancer	A Study of Bevacizumab (Avastin) in Combination with Chemotherapy in Participants with Metastatic Cancer of the Colon or Rectum
NCT00121836	Capecitabine Bevacizumab	Breast Cancer	A Study of Xeloda (Capecitabine) in Women with HER2-Negative Metastatic Breast Cancer
NCT02248571	Bevacizumab Capecitabine Everolimus Exemestane Other: Patient questionnaires	Breast Cancer Recurrent	Patient Preference for Everolimus in Combination with Exemestane or Capecitabine in Combination with Bevacizumab (IMPROVE)
NCT01094184	Bevacizumab Paclitaxel Docetaxel	Breast Cancer	A Study of Bevacizumab with Taxane Therapy in Participants with Triple-Negative Breast Cancer
NCT01695772	5-FU based doublet chemotherapy Bevacizumab	Colorectal Cancer	A Study of Bevacizumab Plus 5-Flurouracil (5-FU) Based Doublet Chemotherapy as Neoadjuvant Therapy for Participants with Previously Untreated Unresectable Liver-Only Metastases from Colorectal Cancer
NCT00577031	Bevacizumab (Avastin) Oxaliplatin Xeloda	Colorectal Cancer	OBELIX Study: A Study of Avastin (Bevacizumab) in Combination With XELOX in Patients With Metastatic Cancer of the Colon or Rectum.
NCT00451906	Platinum-based chemotherapy Bevacizumab (Avastin)	Non-Squamous Non-Small Cell Lung Cancer	A Study of Avastin (Bevacizumab) in Combination with Platinum-Containing Chemotherapy in Patients with Advanced or Recurrent Non-Squamous Cell Lung Cancer
NCT01588990	Oxaliplatin Capecitabine Bevacizumab Leucovorin 5-Fluouracil Drug: Irinotecan	Colorectal Neoplasms	A Translational Study of Bevacizumab in Participants with Metastatic Colorectal Cancer
NCT00793871	Sunitinib Malate (SU011248)	Gastrointestinal Neoplasms, Gastrointestinal Stromal Tumors	Safety and Efficacy Study of Sunitinib Malate In Chinese Patients With Imatinib-Resistant Or -Intolerant Malignant Gastrointestinal Stromal Tumor
NCT02460380	Vitamin D3 Other: Placebo	Polycystic Ovary Syndrome Vitamin D Deficiency	The Effects of Vitamin D on Angiogenic Factors in Women with Polycystic Ovary Syndrome
NCT01206764	Everolimus	Renal Cell Carcinoma	A Trial of Everolimus in Patients with Advanced Renal Cell Carcinoma.
NCT01731886	Procedure: autologous peripheral blood stem cell transplant Lenalidomide Dexamethasone Procedure: stem cell collection Melphalan G-CSF Cyclophosphamide Mesna	Multiple Myeloma	Lenalidomide and Dexamethasone With/Without Stem Cell Transplant in Patients with Multiple Myeloma
NCT02953938	Biological: Ranibizumab Radiation: grid and direct short pulse laser photocoagulation	Macular Edema Secondary to Branch Retinal Vein Occlusion (BRVO)	Study to Show a Superior Benefit in Terms of Reduction of Ranibizumab Injections in Patients Receiving Ranibizumab Plus Laser Photocoagulation Combination Therapy Without Loss of Efficacy and Safety
NCT00706706	Sunitinib Malate (SU011248)	Carcinoma, Renal Cell	Safety and Efficacy Study of Sunitinib Malate as First-Line Systemic Therapy In Chinese Patients With Metastatic Renal Cell Carcinoma
NCT00022516	Cyclophosphamide Methotrexate	Breast Cancer	Low-dose Oral Cyclophosphamide and Methotrexate Maintenance for Hormone Receptor-Negative Early Breast Cancer
NCT00094055	AG013736	Thyroid Neoplasms	Study of the Antiangiogenesis Agent AG-013736 in Patients with Metastatic Thyroid Cancer
NCT01105533	PF-00337210	Neoplasm	A Dose-Finding Study of a New Medication, PF-00337210, That Will Possibly Decrease Blood Supply to Tumors
NCT00140556	Radiation: Chemoradiotherapy Cisplatin Bevacizumab Erlotinib	Head and Neck Cancer Pharynx Cancer	Angiogenic and EGFR Blockade with Curative Chemoradiation for Advanced Head and Neck Cancer

**Table 2:** Types of nanoparticles utilizing angiogenesis pathways.

Nanoparticle Types	Targeting Ligand	Targeted Tumor	Therapeutic/Diagnostic or Both	Imaging Technique Used	Results	References
Iron oxide nanoparticles	A tumor-penetrating peptide, iRGD	Glioblastoma (GBM)	Both	MRI	1- The iron oxide component of the nanoparticles enabled imaging of GBM tumors in mice. 2- Systemic treatment of nanoparticular-bearing GBM mice eradicated most tumors in a GBM animal model and slightly slowed the growth of tumors in another model. 3- The combination of nanoparticles with a tumor-penetrating peptide increased therapeutic efficacy further.	[122]
Gold nanoparticles (AuNPs)	Recombinant human endostatin (rhES)	Metastatic colorectal cancer (mCRC)	Therapeutic	____	1- AuNPs normalized vasculature by promoting vessel stability, indicated by increasing pericyte expression and reducing VEGFR2 in mCRC xenografts. 2- rhES-AuNPs interrupted AGR2-induced vascular formation in HUVECs. These findings suggest that rhES-AuNPs might normalize vessels by interfering with AGR2-mediated angiogenesis in mCRC.	[123]
Hollow mesoporous silica nanoparticles (HMSN NPs)	Macrocyclic chelator, NOTA, PEGylated, and nanoconjugate were attached with (cRGDyK) and radiolabeled with 64Cu for PET imaging.	Glioblastoma	Both	positron emission tomography “PET”	1- Progressing synthesized HMSN-based nanoconjugates that can be used not only to image PET integrin αvβ3 but also for the supply of chemical-therapeutic drugs to carcinogenic lesions for tumor vasculature. 2- Tumor-targeting ability of cRGDyK-conjugated nanoconstructs was significantly enhanced in integrin αvβ3-overexpressing U87MG tumor models by integrin αvβ3-mediated active targeting as well as the EPR effect. 3- In U87MG tumor-bearing mice, a model hydrophobilic anti-carcinogenic (SUN) drug was loaded on high-capability (>400 mg/g) HMSNs, which improved in vivo delivery.	[124]
Liposomal nanoparticles (ICAM-Lcn2-LPs NPs)	Intercellular adhesion molecule-1 (ICAM-1) antibodies, Lcn2 siRNA- encapsulating liposome (ICAM-Lcn2-LP)	Triple-negative breast cancer (TNBC)	Both	____	1- Synthesized ICAM-1-targeted Lcn2 siRNA-encapsulating liposomes significantly suppress in vitro and in vivo angiogenic activities of TNBC cells. 2- Liposomal nanocarriers have both imaging tools and medicinal molecules on a scalable basis. 3- Two kinds of human endothelial cells were used to observe the antiangiogenic properties of ICAM-Lcn2-LP, as seen in reductions in the development and migration of TNBC-mediated endothelial cells (HMVECs and HUVECs).	[125]
Vanadium pentoxide nanoparticles (V_2_O_5_ NPs)	Ethylene glycol	Melanoma	Therapeutic	____	1- The use of V2O5 NPs with C57BL6/J mice dramatically improved their survival relative to untreated mouse controls, demonstrating the therapeutic ability of nanoparticles against melanoma. 2- V2O5 NPs impaired and inhibited blood vasculature differentiation and movement of endothelial cells (HUVECs and EA.hy926) in chick embryos, demonstrating antiangiogenic properties. 3- There was no toxic activity in mice at subchronic exposure to V2O5 NPs with in-vivo toxicity analysis.	[126]
PEG-PLA nanoparticles NPs	APT_EDB_	Glioma	Therapeutic	____	1- PTX-loaded APT-NPs indicated satisfactory encapsulated efficiency, loading capacity, and size distribution. 2- In both subcutaneous and intracranial xenograft models, APT-NP-PTX demonstrated increased antiglioma potency over unmodified nanoparticles and Taxol^®^. 3- APT-NPs achieved much higher and precise aggregation within glioma after IV administration, as both in-vivo animal imaging and tissue dissemination analyses have shown.	[127]
Cuprous oxide nanoparticles (CO-NPs)	Nontargeted ligand	____	Therapeutic	____	1- CO-NPs are able to cause improvements in cell morphology and in vitro or in vivo doses to prevent cell proliferation, migration, and tube forming. 2- CO-NPs have been shown to inhibit dosage and time of expression based on protein and mRNA levels, but they have little impact on the expression of VEGF or VEGFR1.	[128]
Cerium oxide “Nanoceria” nanoparticles (NCe NPs)	Nontargeted ligand	Ovarian cancer	Therapeutic	____	1- Nanocerides (NCes) were crafted from cerium oxide NPs with antioxidant properties for use as a therapeutic agent in ovarian cancer. 2- NCes blocked mediated VEGF165 in human endothelial umbilical vascular cells, capillary tube development, activation of VEGFR2, and MMP 2 (HUVEC). 3- Reduction in tumor mass, as noted by a decreased CD31 stain and specific apoptosis of vascular endothelial cells, followed by a mitigation of angiogenesis.	[129]
Vitamin E “TPGS” micellar nanoparticles	Styrene-maleic acid (SMA)	Renal cell carcinoma	Therapeutic	____	1- CFM-4.1 encapsulated in TPGS-based vitamin E nanomicelles, leading to a higher loading CFM-4.16 and water-soluble formulation (30% *w*/*w*). 2- The formula of CFM-4.16 prevented the in-vitro and suppressed development of parental A498 RCC cell xenografts by inducing apoptosis of parent RCC cells in vitro and everolimus-resistant cells.	[130]
Silver nanoparticles (Ag NPs)	Nontargeted ligand	Breast cancer cell line MCF7	Therapeutic	____	1- Ag NPs are inhibited by HIF-1α and its aggregation of proteins and downstream target expression in MCF7 cell development. 2- Ag NPs work to suppress the action of HIF-1α in cells under hypoxic conditions, leading to VEGF-A and GLUT1 downregulation and inhibition of angiogenesis.	[131]
Zinc oxide nanoparticles (ZnO NPs)	Gelatin biopolymers	Liver cancer cell line (HePG2)	Therapeutic	____	1- Ge-ZnO NPs inhibited the viability of HepG2 cell lines; in addition, Ge-ZnO NPs and zinc acetate showed antiangiogenesis activity in chick embryos. 2- The findings for the CAM test showed that in chick embryos, antiangiogenesis was higher than in biopolymer gelatine for Ge ZnO NPs and zinc acetate. 3- HepG2 cells treated with 100 µg/mL Ge-ZnO NPs showed ruptures and a consequent loss of membrane integrity.	[132]
W_18_O_49_ nanoparticles	anti-HER-2 monoclonal antibody	Breast cancer	Both	CT	1- In vivo research verified that WOHA NPs could precisely mark metastatic HER-2 lymph nodes and exclude laser irradiation from cancer cells. 2- WOHA NP-made PTT could prolong the survival rate of breast-bearing mice by inhibition of cancer cell metastases in animals 3- In mice with HER-2 positive metastases, a simple distinction can be made between lymph nodes under CT guidance; laser ablation can selectively remove them.	[133]
Bismuth-based nanoparticles (Bi_2_S_3_ NPs)	Hyaluronic acid (HA)	Solid tumors	Both	CT	1- Not only was the intrinsic radioactivity in cancer cells enhanced in HA-Bi2O3 NPs encapsulated with Bi atoms through absorption of high-energy photons and the emission of secondary electrons and Auger electrons, but it also had high ray attenuation coefficients in favor of CT-imaging-guided radiotherapy, which had a substantial increase in radioactivity. 2- HA-Bi2O3 NPs were especially suited to the overexpression of CD44 receptors, possessing favorable water solubility and excellent biocompatibility.	[134]
Selenium nanoparticles (Se NPs)	Sulforaphane	Breast, colon, prostate cancers	Therapeutic	____	1- The cell growth inhibitory effect between SFN and SeNPs was highly synergistic in all cancer cell lines. 2- Important high selectivity has been observed between cancer and normal cells. Cytotoxicity is several times smaller in human cells than in cancers.	[135]
Carbon allotrope nanoparticles: Ultra-dispersed detonation diamond (UDD) and microwave-radiofrequency (MW-RF) carbon allotrope	Nontargeted ligand	Glioblastoma	Therapeutic	____	1- Nanoparticles of UDD and MW-RF decrease tumor mass and volume and block the production of new blood vessels in in-vivo GBM tumors. 2- UDD NP was found to decrease the expression of FGF-2 and VEGF substantially, while MW-RF NP decreased the expression of VEGF only.	[136]

## Data Availability

Not applicable.
